# GFP fusions of Sec-routed extracellular proteins in *Staphylococcus aureus* reveal surface-associated coagulase in biofilms

**DOI:** 10.15698/mic2023.07.800

**Published:** 2023-06-28

**Authors:** Dominique C. S. Evans, Amanda B. Khamas, Lisbeth Marcussen, Kristian S. Rasmussen, Janne K. Klitgaard, Birgitte H. Kallipolitis, Janni Nielsen, Daniel E. Otzen, Mark C. Leake, Rikke L. Meyer

**Affiliations:** 1 Interdisciplinary Nanoscience Center, Aarhus University, Aarhus, Denmark.; 2 Department of Physics, University of York, York, UK.; 3 Department of Biochemistry and Molecular Biology, University of Southern Denmark, Odense, Denmark.; 4 Department of Biology, University of York, York, UK.; 5 Department of Biology, Aarhus University, Aarhus, Denmark.

**Keywords:** fusion protein, Gram positive bacteria, monomeric superfolder GFP, coagulase, biofilms

## Abstract

*Staphylococcus aureus* is a major human pathogen that utilises many surface-associated and secreted proteins to form biofilms and cause disease. However, our understanding of these processes is limited by challenges of using fluorescent protein reporters in their native environment, because they must be exported and fold correctly to become fluorescent. Here, we demonstrate the feasibility of using the monomeric superfolder GFP (msfGFP) exported from *S. aureus.* By fusing msfGFP to signal peptides for the Secretory (Sec) and Twin Arginine Translocation (Tat) pathways, the two major secretion pathways in *S. aureus,* we quantified msfGFP fluorescence in bacterial cultures and cell-free supernatant from the cultures. When fused to a Tat signal peptide, we detected msfGFP fluorescence inside but not outside bacterial cells, indicating a failure to export msfGFP. However, when fused to a Sec signal peptide, msfGFP fluorescence was present outside cells, indicating successful export of the msfGFP in the unfolded state, followed by extracellular folding and maturation to the photoactive state. We applied this strategy to study coagulase (Coa), a secreted protein and a major contributor to the formation of a fibrin network in *S. aureus* biofilms that protects bacteria from the host immune system and increases attachment to host surfaces. We confirmed that a genomically integrated C-terminal fusion of Coa to msfGFP does not impair the activity of Coa or its localisation within the biofilm matrix. Our findings demonstrate that msfGFP is a good candidate fluorescent reporter to consider when studying proteins secreted by the Sec pathway in *S. aureus*.

## INTRODUCTION

Green fluorescent protein (GFP) has been used for decades as an intracellular reporter for gene expression and as a fluorescent tag to visualise single proteins in the cytoplasm of bacteria [1]. An advantage of fluorescent proteins is that samples do not need to be stained and incubated to visualise the protein. GFP and other fluorescent proteins have therefore been instrumental for studies into protein localisation, visualising subcellular compartments, monitoring gene expression, tissue labelling, as well as DNA and RNA labelling [2].

While GFP fusion proteins have taught us much about intracellular proteins, little research has been done on extracellular proteins, such as surface-bound proteins or other secreted proteins. Some GFP variants have been successfully secreted to the periplasm and outer membrane of Gram-negative bacteria [3–5], however, there are only few examples of this for Gram-positive bacteria. To our knowledge, GFP secretion in Gram-positive bacteria has only been achieved in a small number of organisms, including *Corynebacterium glutamicum* [6], *Bacillus subtilis* [7, 8], *Streptococcus mutans* [9], *Mycobacterium smegmatis* [10], and *Staphylococcus epidermidis* [11]. Split GFP has additionally been successfully secreted by *B. subtilis* [12]. There is a multitude of reasons why generation of GFP-fusion proteins may fail. In particular, the fusion protein may not be successfully secreted, the GFP may misfold and fail to become fluorescent in the extracellular environment, or the chromophore may not mature properly [6, 13]. Additionally, the level of transcription and translation, protein turnover rate, and photobleaching further complicate imaging of GFP fusions [13].

Most extracellular proteins are secreted in an unfolded state via the Secretory (Sec) pathway, where they are exported across the cytosolic membrane into the periplasm in Gram-negative bacteria or outside the cell in Gram-positive bacteria [14]. It is a highly conserved pathway present in all classes of bacteria [15]. Sec-routed proteins have a signal peptide at their N-terminus that directs them towards the SecYEG membrane protein channel, after which they are driven stepwise across the membrane by the ATPase molecular motor SecA [16]. The transported protein then folds on the trans side of the membrane. In many Gram-negative bacteria, SecB stabilises and targets the unfolded protein to SecA, while in Gram-positive and other Gram-negative bacteria, general chaperones maintain the protein in an unfolded state [16]. Another common secretion pathway is the Twin Arginine Translocation (Tat) pathway, in which proteins are exported in a folded state [17], however, not all bacterial species have a Tat pathway [18]. Tat-routed proteins have an N-terminal signal sequence containing a twin-arginine motif that gives the pathway its name [19]. Some proteins that are secreted through the Tat-pathway, such as proteins with co-factors that bind to cytoplasmic proteins, usually need to fold in the cytoplasm to function correctly [20]. The Tat pathway contains three subunits TatA, TatB, and TatC in Gram-negative bacteria and two subunits TatA and TatC in Gram-positive bacteria, which bind the signal peptide and form a membrane spanning channel [15]. These subunits have been studied previously using fluorescent protein reporters in live *Escherichia coli* cells [21]. Folded proteins are exported outside of the cell in Gram-positive bacteria, and to the periplasm in Gram-negative bacteria, where they may be exported across the outer membrane via other mechanisms [15].

The aims of our present study were to investigate whether monomeric superfolder GFP (msfGFP) is a good candidate for extracellular fusion proteins in *Staphylococcus aureus*, and to determine if msfGFP can be secreted by either of the two secretion pathways Sec and Tat. *S. aureus* is a Gram-positive coccus which has both the Sec and Tat secretion pathways [15, 18]. It is a major biofilm-forming human pathogen that can cause skin and soft tissue infections, endocarditis, osteomyelitis, and toxic shock syndrome [22]. *S. aureus* utilises many surface-associated and secreted proteins to interact with host tissue, to establish infections, and evade the immune system [23]. These proteins include a family known as microbial surface components recognising adhesive matrix molecules (MSCRAMMs), all of which contain a Sec signal peptide [23]. Examples include clumping factors A and B (ClfA and ClfB) that clump bacteria by binding host fibrinogen and aid tissue colonisation [24], fibronectin binding proteins A and B (FnBPA and FnBPB) that bind host fibronectin, fibrinogen, and elastin, and therefore facilitate attachment to host tissues via host proteins [24], and collagen adhesin (Cna) that facilitates attachment via collagen and helps *S. aureus* escape immune cells [23]. *S. aureus* also secretes a family of proteins called secretable expanded repertoire adhesive molecules (SERAMs). These include extracellular adherence protein (Eap), extracellular matrix protein-binding protein (Emp), extracellular fibrinogen binding protein (Efp), coagulase (Coa), and von Willebrand factor binding protein (vWbp). Eap inhibits neutrophils and therefore inhibits the immune response [25], Emp binds host fibronectin, fibrinogen, and vitronectin [26], which appears to be important for virulence [26], and Efp inhibits phagocytosis [27] and decreases wound healing [28]. Coa and vWbp bind to and activate host prothrombin to hijack the host coagulation cascade and thereby trigger the formation of fibrin fibers, a major component of the biofilm extracellular matrix [29], in two concentric structures: a cell surface-associated pseudocapsule and an extended outer network, which together act as mechanical barriers against immune attack [30], enhance virulence [31], and increase adhesion to surfaces [32]. *S. aureus* would benefit from a reliable system with which to label and visualise proteins such as these that are important to its virulence and pathogenicity, especially in complex environments such as biofilms where traditional antibody labelling methods may fail. Antibodies are approximately 10 nm in size [33], which is relatively large compared to many matrix components, such as DNA which has a width of approximately 2.5 nm and many proteins which are less than 10 nm in size. Therefore, antibodies may fail to penetrate some biofilm matrices and fail to label them correctly.

We chose msfGFP as our model fluorescent protein due to its brightness and enhanced folding properties [34], and it has been previously shown to fold in traditionally challenging environments such as the periplasm of Gram-negative bacteria [34]. We investigated Sec- and Tat-secreted msfGFP by fusing msfGFP to Sec and Tat signal peptides in overexpression plasmids and subsequently measuring the increase in fluorescence from bacterial cultures and cell-free culture supernatants. After confirming that msfGFP is suitable to visualise secreted proteins, we developed a C-terminal chromosome-integrated fusion between msfGFP and Coa in *S. aureus*, which is predicted to have a Sec-type signal peptide [35]. We demonstrated that fusion to msfGFP did not impair the biological function of Coa, and that Coa:msfGFP fusion proteins revealed the location of Coa in *S. aureus* biofilms. Coa is responsible for producing a fibrin pseudocapsule and has previously been located within the pseudocapsule [30, 31]. We demonstrate that Coa localises to cell surfaces, where we hypothesise that it associates with the cell to facilitate fibrin production near the surface of bacteria.

## RESULTS

### msfGFP is secreted via Sec and becomes fluorescent in the extracellular environment

**Figure 1:**
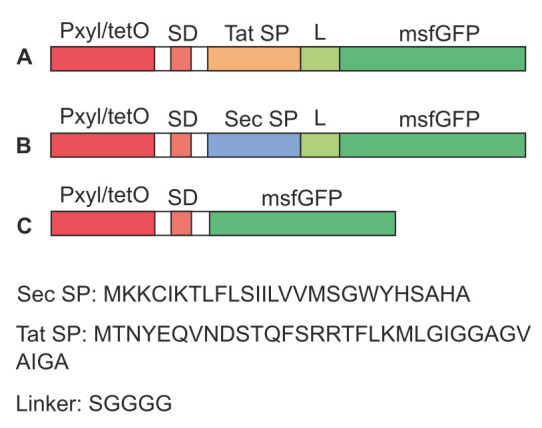
FIGURE 1: Visual schematics of constructs expressing fusion proteins under control of the inducible P_xyl/tetO_ promoter in pRMC2. **(A)** Tat:msfGFP, **(B)** Sec:msfGFP, and **(C)** msfGFP control. SD = Shine-Dalgarno sequence, SP = signal peptide, and L = linker. Amino acid sequences for the Tat signal peptide [18], Sec signal peptide [45] and linker are given in the figure. DNA sequences are provided in Supplementary S1.

The fluorescent protein msfGFP is a good candidate for tagging extracellular proteins in Gram-positive bacteria, but its implementation depends on whether it can be secreted and fold properly in the extracellular space. We therefore tested the ability of msfGFP to become fluorescent after secretion via the Tat and Sec pathways in *S. aureus*. We generated four strains of *S. aureus* that carried different variants of the overexpression pRMC2 plasmid (**Figure 1**). Strain 1 contained an empty pRMC2 vector, which served as negative control, strain 2 contained pRMC2 encoding msfGFP without a signal peptide and was used as a positive control to verify msfGFP expression, strain 3 contained pRMC2 encoding Tat:msfGFP for secretion of GFP through the Tat pathway, and strain 4 contained pRMC2 encoding Sec:msfGFP for secretion through the Sec pathway. The presence of functional msfGFP was then measured as the appearance of green fluorescence of cultures and cell-free supernatants using a fluorescence plate reader after inducing expression of msfGFP from the plasmid.

**Figure 2:**
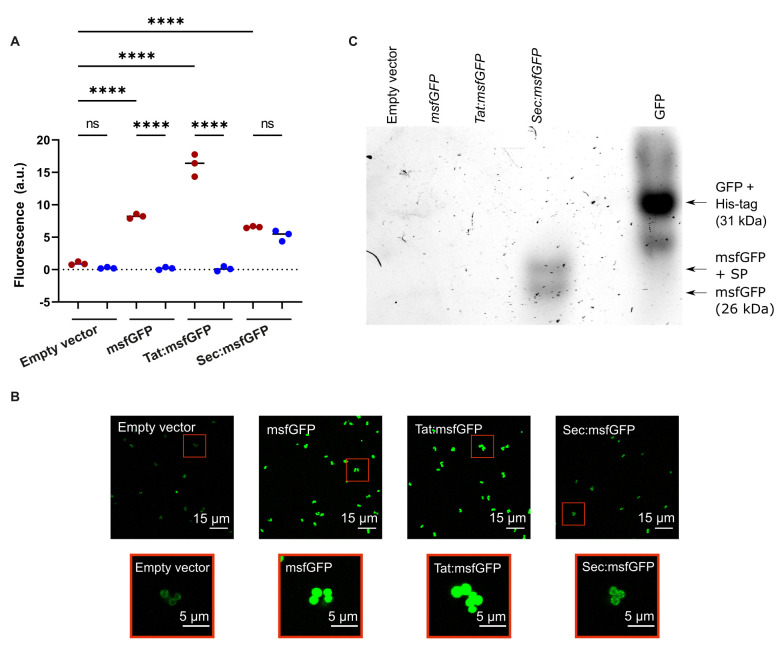
FIGURE 2. **(A)** Fluorescence intensity from excitation of msfGFP in cell cultures (red circles) or cell-free supernatants (blue circles) of *S. aureus* expressing msfGFP from the pRMC2 vector. msfGFP was fused to either Tat or Sec signal peptides, no signal peptide, or not expressed at all (empty vector). Black bars indicate group medians. Samples were compared using a one-way ANOVA followed by a Tukey's test; **** denotes a p < 0.0001 significance level and ns denotes no significance. **(B)** CLSM images of *S. aureus* cells expressing msfGFP fusions. Red boxes indicate zoomed in images. All fluorescence images had their brightness increased equally using Fiji ImageJ for clear visualisation. **(C)** In-gel fluorescence of GFP/msfGFP in a native PAGE gel containing supernatants from cultures expressing the empty pRMC2 vector, msfGFP without a signal peptide, or fused to either a Sec or Tat signal peptide.

Only the culture expressing Sec:msfGFP produced fluorescence in the cell-free supernatant, which indicated that msfGFP can secrete and fold correctly when exported by the Sec-pathway (**Figure 2A**). The fluorescence intensity from the culture (bacteria and supernatant) was at a similar level to the supernatant alone, indicating that msfGFP was primarily present in the supernatant. In cultures expressing Tat:msfGFP or msfGFP without a signal peptide, fluorescence was detected in bacterial cultures but not the supernatants (**Figure 2A**), indicating that msfGFP could fold correctly within cells, but was not secreted via the Tat pathway. Although Tat:msfGFP was not successfully secreted, the fluorescence intensity from Tat:msfGFP cell cultures was higher than the fluorescence intensity from Sec:msfGFP cell cultures, which may reflect differences in the activity of the two different pathways, different rates of msfGFP transcription, translation, or protein folding when fused to a particular signal peptide.

The presence of fluorescent msfGFP in the intracellular and extracellular environment was verified by CLSM imaging of cell cultures expressing Tat:msfGFP, Sec:msfFP, msfGFP, and cells containing the empty vector. As expected, msfGFP fluorescence was detected inside cells expressing Tat:msfGFP and msfGFP (**Figure 2B**). There was a weak fluorescence in *S. aureus* expressing Sec:msfGFP, which reflects that there was a small fraction of GFP that was not secreted from the bacteria or that remained linked to the cell wall. Furthermore, in-gel fluorescence showed that only the Sec:msfGFP strain secreted a functional msfGFP (**Figure 2C**). However, the secreted msfGFP was found in two distinct sizes in the supernatant of the Sec:msfGFP cultures, which indicates that the signal peptide was not always removed from some of the msfGFP during secretion. There is an additional band underneath the GFP control (**Figure 2C**), which is likely GFP that lacks a His-tag. Although we have confirmed msfGFP is found in the supernatant by bulk measurements using a plate reader (**Figure 2A**), the fluorescence could not be seen in the supernatant using CLSM because the fluorescent protein was too diluted to be visualised. There is also a weak surface-associated fluorescent signal seen with CLSM on *S. aureus* containing an empty vector without msfGFP, which is due to autofluorescence from ATc [36].

### Coa:msfGFP produces a functional coagulase that localises within the fibrin pseudocapsule

msfGFP was successfully secreted via the Sec pathway, so to demonstrate its suitability to tag extracellular proteins, it was fused to Coa by insertion into the *S. aureus* chromosome via allelic exchange. Coa is one of two coagulases that hijack the human coagulation cascade and triggers the formation of a fibrin network around *S. aureus* cells, a major component of the biofilm extracellular matrix *in vivo,* that protects *S. aureus* from the host immune system during infection [29, 30]. In order to confirm that the chromosome-integrated *coa:msfGFP* had not impacted the ability of Coa to cause coagulation, the fusion protein was first created in a mutant strain that lacks the other coagulase: Von Willebrand factor binding protein (vWbp) [37]. Loss of function of coagulase would then result in inability to coagulate plasma.

**Figure 3:**
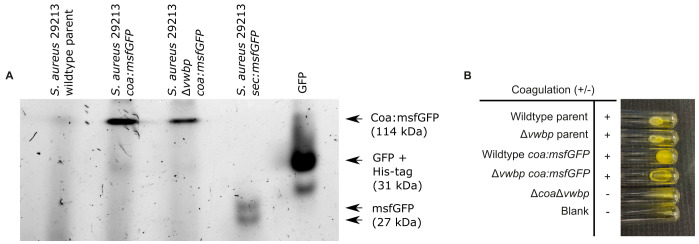
FIGURE 3: The Coa:msfGFP fusion protein was successfully secreted from *S. aureus* and functioned correctly. **(A)** n-gel fluorescence of GFP/msfGFP in a native PAGE gel containing supernatants from the S. aureus wildtype parent strain, as well as S. aureus wildtype and S. aureus Δvwbp both expressing Coa:msfGFP. His-tagged GFP and supernatant from the strain expressing Sec:msfGFP were also loaded to the gel to serve as a molecular marker and as a positive control for GFP and msfGFP fluorescence, respectively. The gel shows that Coa:msfGFP was secreted as an intact, fluorescent protein, giving a band at the expected weight of msfGFP and Coa combined [53]. **(B)** Coagulation of *S. aureus* 29213 wildtype and Δ*vwbp* producing either Coa:msfGFP or unmodified Coa and *S. aureus* 29213 Δ*coa*Δ*vwbp* after 24 hours incubation with human plasma at 37°C. All strains producing Coa coagulated plasma, while the double mutant Δ*coa*Δ*vwbp* did not.

**Figure 4:**
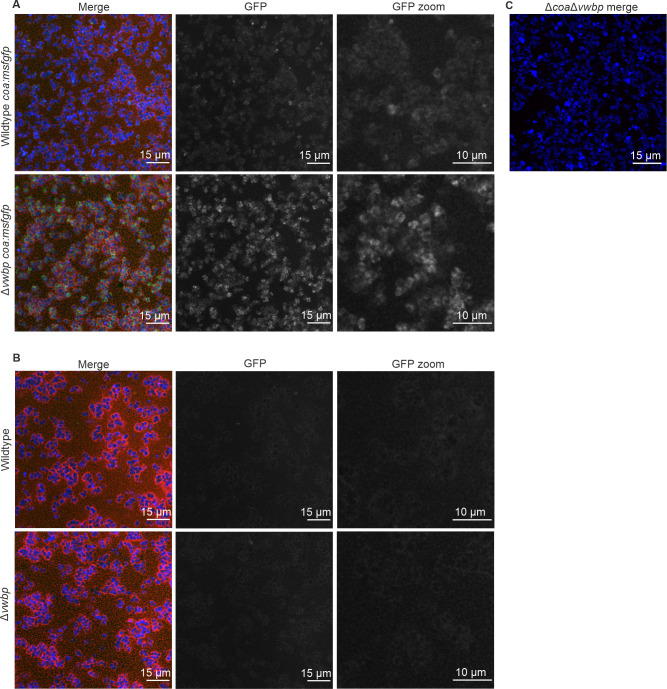
FIGURE 4. **(A)** CLSM images of *S. aureus* wildtype and *S. aureus* Δ*vwbp* biofilms producing Coa:msfGFP. The composite image (left) is displayed along with the channel containing only signal from Coa:msfGFP (middle) and a zoomed in image of that channel (right). Coa:msfGFP localised to the surface of bacteria within the fibrin pseudocapsule. **(B)** The parental strains of *S. aureus* that produce unmodified Coa when imaged with the same imaging settings as modified bacteria producing Coa:msfGFP. No fluorescence was detected, which confirms that the fluorescence in Figure 4A originates from msfGFP and not from autofluorescence. **(C)** A double mutant lacking both Coa and vWbp (Δ*coa*Δ*vwbp*) forms no fibrin at all. Biofilms were grown in BHI containing 50% human plasma for 2 h. Bacteria (wildtype and Δ*vwbp*) were visualised by staining with SYTO 41 (blue), fibrin was visualised by amending Alexa 647-conjugated fibrinogen to media (red), and Coa was visualised by fluorescence emitted from msfGFP (green). The double mutant expressed *gfp* from a plasmid pCM29 [54] (blue), and fibrin was also visualised by addition of Alexa-647-conjugated fibrinogen to the media (red). Brightness for each colour channel for each image were increased equally using Fiji ImageJ to visualise the data for both (A) and (B).

The fusion protein Cos:msfGFP was secreted successfully from *S. aureus* and the fusion protein did not get cleaved, demonstrated by in-gel fluorescence analysis (**Figure 3A**). Fluorescence from GFP was present in the supernatant of bacterial cultures expressing Coa:msfGFP and not in cultures without Coa:msfGFP, demonstrating that the fusion protein was secreted extracellularly (**Figure 3A**). Coa:msfGFP from the supernatant of bacterial cultures did not travel as far through the gel as GFP alone, or msfGFP fused to a Sec signal peptide. Therefore, the weight of the fusion protein was much larger, demonstrating that the protein is intact and contains both Coa and msfGFP (**Figure 3A**). Coa was also functional, as *S. aureus* with chromosome-integrated *coa:msfGFP* coagulated plasma similarly to the parental strains (**Figure 3B**), and biofilms formed similar fibrin structures as the parental strains, i.e. fibrin was visible as pseudocapsules surrounding clusters of bacteria and as an extended fibrous network between clusters of bacteria (**Figure 4A, 4B)**). This was true for both the wildtype and the mutant lacking vWbp, thus the fusion to msfGFP did not inhibit the function of Coa. We confirmed that coagulation occurred due to Coa and vWbp alone by including a control mutant of *S. aureus* that lacks both *coa* and *vwbp*, which did not coagulate plasma (**Figure 3B**) nor produce fibrin fibers in the biofilm matrix (**Figure 4C**). Fibrin was visualised by the addition of fluorescently labelled fibrinogen to the biofilm growth medium, which is converted into fibrin fibers by an activated complex formed by Coa and vWbp binding to host prothrombin. The small amount of red fluorescence seen in **Figure 4C** are aggregates of fluorescent fibrinogen that have not been converted into fibrin because of the absence of both Coa and vWbp.

To assess the location of Coa in *S. aureus* biofilms, we visualised the bacterial cells, fibrin, and Coa:msfGFP by CLSM. Coa:msfGFP localised to the surface of the bacteria, where we predicted that Coa catalyses the formation of a fibrin pseudocapsule (**Figure 4A**). This finding corroborates previous studies, which also detected Coa in the fibrin pseudocapsule by immunolabelling [30]. Biofilms of the parental strain were used as negative controls, and here we detected no fluorescence from GFP (**Figure 4B**). We have thus demonstrated the use of msfGFP for labelling a protein secreted by the Sec pathway in *S. aureus*. The signal from msfGFP appears brighter in the mutant lacking vWbp, and this is most likely caused by a less dense extracellular matrix in this strain which lacks one of the coagulases, and the signal from msfGFP is therefore attenuated less.

## DISCUSSION

We show that msfGFP can be used to generate extracellular fluorescent fusion proteins in *S. aureus*, but that the application is limited to proteins that are secreted through the Sec pathway. When fused to Coa, msfGFP did not hinder the biological function of Coa, and the fusion protein localised to the fibrin pseudocapsule surrounding clusters of *S. aureus* cells. This result is in agreement with previous studies [30, 31] and indicates that fusion to msfGFP does not cause Coa to mislocalise or malfunction. Therefore, msfGFP is a good candidate for tagging *S. aureus* proteins exported by the Sec pathway, and the majority of extracellular proteins are indeed secreted by this pathway [14].

msfGFP has a superfolding mutation that makes it fold quickly and without chaperones, even when fused to another protein, and it exhibits a high level of brightness [34] that makes it ideal for creating fusion proteins in the extracellular environment. Correct folding is essential for chromophore formation and fluorescence, while fast folding is also important for the protein to fold into its 3D conformation in time to avoid cleavage by extracellular proteases that clear unfolded or misfolded proteins away from the cell surface. msfGFP is also monomeric, which makes it less likely to aggregate and cause artefacts, which makes it a good candidate for many fusion proteins. We have demonstrated for the first time the generation of fluorescent fusion proteins for a secreted protein in *S. aureus*, and this approach now opens possibilities of studying the location of secreted proteins that remain associated with the extracellular matrix of staphylococcal biofilms. The fluorescence signal was fairly dim when imaging Coa:msfGFP, however, it is not known how much Coa is produced and therefore the concentration could be low. Additionally, we imaged the fusion protein in the complex environment of a biofilm. Biofilms are thick, heterogeneous samples that are autofluorescent and attenuate and distort both the excitation and emission from fluorescent molecules, which makes them a challenging environment to image in. However, the fact that Coa:msfGFP could be visualised by standard CLSM imaging is encouraging, and advanced microscopes with more sensitive detection will facilitate more detailed analyses. For example, single-molecule microscopy on live bacteria has revealed important details of Tat-mediated transport in Gram-negative *E. coli*, and similar investigations could be pursued for Sec-secreted proteins in Gram-positive bacteria using msfGFP fusions [21]. In particular, future studies could utilise total internal reflection fluorescence (TIRF) microscopy to investigate extracellular secretion between the cell and a surface such as an agarose pad or glass coverslip, that are sensitive at single-molecule GFP detection limits in live bacteria [38, 39], or faster millisecond Slimfield microscopy that could potentially enable mobility studies of extracellular secreted components [40–42].

*S. aureus* is a major biofilm-forming human pathogen that establishes infections, causes disease, and evades the immune system through a number of secreted and cell surface associated proteins, many of which contain a Sec-type signal peptide [23]. Fusions with msfGFP will greatly benefit future research into these proteins. We do not know whether msfGFP would be exported correctly via the Sec pathway in other bacterial species; past studies into GFP export via the Tat pathway in Gram positive bacteria revealed that a different GFP variant was not exported correctly in all species tested [6]. The authors speculated that their results were due to differences in the physical or chemical structure of the cell wall, or in the quality control mechanisms of the Tat translocases. Such interspecies differences may also affect the outcome when using msfGFP for Sec exported proteins, and this is important to bear in mind. The assay performed by expression of msfGFP from pRMC2 vector in our study, however, provides an easy tool for checking the feasibility of msfGFP secretion in other *Staphylococci* that are compatible with this vector, and the Tat and Sec signal peptide constructs can be cloned into different vectors for studies in other Gram-positive bacteria.

We have confirmed that msfGFP is a good candidate for labelling proteins secreted by the Sec pathway in *S. aureus*. We fused *msfGFP* to *coa* in the *S. aureus* chromosome and demonstrated that fusion to msfGFP did not prevent Coa from functioning correctly, that msfGFP could fold correctly and fluoresce in the extracellular environment, and that the fusion protein localised as expected in the extracellular environment. *S. aureus* utilises a myriad of surface associated and secreted proteins to establish infections and cause disease, and our work opens the door for developing fusion proteins to investigate these and progress our understanding of *S. aureus* infection.

## MATERIALS AND METHODS

### Materials, bacterial strains, and growth conditions

All bacterial strains, plasmids, and primers used are listed in **Table 1**. For long-term storage, bacteria were stored in 25% glycerol at -80°C. *E. coli* and *S. aureus* were cultured in Luria Broth (LB, L3522, Sigma-Aldrich) and Brain Heart Infusion (BHI, 53286, Millipore), respectively, at 37°C with 180 rpm shaking. When grown on agar, 15 g/L agar (A1296, Sigma-Aldrich) was added to the media. For plasmid selection, the media was supplemented with 25 µg/ml or 10 µg/ml chloramphenicol (Cm, C0378, Sigma-Aldrich), or 100 µg/ml ampicillin (Amp, A9393, Sigma-Aldrich). Biofilms were grown in modified BHI (mBHI) supplemented with 50% heparin stabilised human plasma to mimic physiological conditions. mBHI is BHI supplemented with 2.1 mM CaCl_2_ (C3881, Sigma-Aldrich) and 0.4 mM MgCl_2_ (31413, Sigma-Aldrich). When low autofluorescence conditions were required, bacteria were suspended in mM9 medium. mM9 is a minimal medium comprising of M9 salts (M6030, Sigma-Aldrich) supplemented with 2 mM MgSO4 (M1880, Sigma-Aldrich), 0.1 mM CaCl_2_ (C3881, Sigma-Aldrich), 1% glucose (1.08346, Merck), 1% casamino acids (Gibco, 223050), 1 mM Thiamine-HCl (T4625, Sigma-Aldrich), and 0.05 mM nicotinamide (72340, Sigma-Aldrich) [43]. Plasma was collected from blood donated by Aarhus University Hospital by centrifugation at 2000 x *g* for 15 minutes at 4°C and stored in aliquots at -80 °C. Before use, frozen plasma was immediately thawed in a water bath at 37°C. Tat and Sec signal peptide fusion protein expression was induced with the addition of 340 ng/ml anhydrotetracycline (ATc, 94664, Sigma-Aldrich).

**Table 1.** Bacterial strains and plasmids used in this study.

**Bacterial Strain**	**Description**	**Reference**
*E. coli* IM08B	mcrA Δ(mrr-hsdRMS-mcrBC) ϕ80lacZΔM15 ΔlacX74 recA1 araD139 Δ(ara-leu)7697 galU galK rpsL endA1 nupG Δdcm ΩPhelp-hsdMS (CC8-2) ΩPN25-hsdS (CC8-1). Derived from *E. coli* K12 DH10B. Deficient in cytosine methylation (Δ*dcm*) and methylates adenine (*hsdMS*) to bypass *S. aureus* restriction barriers	[49]
*S. aureus* 29213	*Staphylococcus aureus* subsp. *aureus* Rosenbach (ATCC29213)	www.atcc.org
*S. aureus* Δ*vwbp*	*Staphylococcus aureus* subsp. *aureus* Rosenbach (ATCC29213) (with *vwbp* gene deleted from the chromosome)	This study
*S. aureus ΔcoaΔvwbp*	*Staphylococcus aureus* subsp. *aureus* Rosenbach (ATCC29213) (with *coa* and *vwbp* genes deleted from the chromosome)	This study
*S. aureus coa:msfGFP*	*Staphylococcus aureus* subsp. *aureus* Rosenbach (ATCC29213) with Coa:msfGFP genomically integrated fusion protein.	This study
*S. aureus* ATCC 29213 Δ*vwbp coa:msfGFP*	*Staphylococcus aureus* subsp. *aureus* Rosenbach (ATCC29213) Δ*vwbp* with Coa:msfGFP genomically integrated fusion protein.	This study
**Plasmid**	**Description**	**Reference**
pRMC2	*E. coli*/*S. aureus* shuttle plasmid with inducible promoter P_xyl/tetO_. Amp^r^, Cm^r^. pRMC2 was a gift from Tim Foster Addgene (http://n2t.net/addgene:68940; RRID:Addgene_68940).	[44]
pUC57-*msfGFP*	*E. coli* plasmid carrying *msfGFP* and *coa:msfGFP*. Amp^r^	Genscript
pIMAY	*E. coli*/Staphylococci temperature sensitive vector for allelic exchange. Cm^r^. Inducible *secY* antisense. pIMAY was a gift from Ian Monk. (Also available at Addgene plasmid # 68939; http://n2t.net/addgene:68939; RRID:Addgene_68939).	[50]
pRMC2-*msfGFP*	pRMC2 with Shine-Dalgarno sequence and *msfGFP* inserted downstream from P_xyl/tetO_ promotor. Optimised for *S. aureus* codon usage. Deposited in Addgene: Plasmid # 194913	This study
pRMC2-*sec:msfGFP*	pRMC2 with Shine-Dalgarno sequence and Sec signal peptide sequence fused to *msfGFP* inserted downstream from P_xyl/tetO_ promotor. Optimised for *S. aureus* codon usage. Deposited in Addgene: Plasmid # 194914	This study
pRMC2-*tat:msfGFP*	pRMC2 with Shine-Dalgarno sequence and Tat signal peptide sequence fused to *msfGFP* inserted downstream from P_xyl/tetO_ promotor. Optimised for *S. aureus* codon usage. Deposited in Addgene: Plasmid # 194915	This study

### Construction of pRMC2 overexpression vector carrying signal peptide:msfGFP constructs

Tat and Sec signal peptide sequences were fused to *msfGFP* to create *tat:msfGFP* and *sec:msfGFP* in the vector pRMC2 (**Figure 1**), a plasmid with an inducible P_xyl/tetO_ promoter and origin of replication for *E. coli* and *Staphylococci* (**Table 1**) [44]. A positive control was also constructed expressing msfGFP with no signal peptide (**Figure 1**). Note that the Shine-Dalgarno sequences were added later as described in the following section.

Sequences for msfGFP [34], Tat [18], and Sec signal peptides [45] were reverse translated with an *S. aureus* USA300 codon usage table (see Supplementary Table S1 for sequences). The RNA polymerase α and β subunits are highly conserved, and their nucleotide sequences were used to predict codon usage in *S. aureus* USA300 and *S. aureus* 29213, and an *S. aureus* USA300 codon usage table was deemed suitable. Signal peptide sequences were ordered as oligos (Thermo Fisher Scientific) and *msfGFP* with a linker at its N-terminal was ordered on a high copy plasmid (pUC57, Genscript). The signal peptide sequences and *msfGFP* were amplified by PCR with Phusion polymerase (F566S, Thermo Fisher Scientific) according to the manufacturer's instructions. The primers (Invitrogen), listed in **Table 2**, contained overhangs intended to join fragments and add KpnI and EcoRI restriction sites at the 5' and 3' ends. Primers 2Ftg/2Rb and 2Fsg/2Ra were used to amplify Tat and Sec signal peptide sequences, respectively, and *msfGFP* was amplified with 1Fa/1Rsg. The signal peptide sequences were joined to *msfGFP* via SOE-PCR to create *tat:msfGFP* (primers 1Fa/Rtg) and *sec:msfGFP* (primers 1Fa/2Ra). *msfGFP* was also amplified alone with no signal peptide sequence to be used as a control. PCR products were analysed by gel electrophoresis and purified with the GenElute Gel Extraction Kit (NA1111, Sigma-Aldrich). All PCR products and pRMC2 were digested by KpnI (FD0524, Thermo Fisher Scientific) and EcoRI (FD0274, Thermo Fisher Scientific), and PCR products were ligated into pRMC2 with T4 DNA ligase (EL0011, Invitrogen) according to the manufacturer's protocols.

**Table 2.** Primers used in this study. Annealing sequence of primers is given in upper case, and overhangs in lower case text.

**Primer**	**Sequence (5′ – 3′) and description**	**Reference**
FwdRMC2	CTCTTCGCTATTACGCCAGC Anneals to pRMC2 multiple cloning site.	This study
RevRMC2	TGGATCCCCTCGAGTTCATG Anneals to pRMC2 multiple cloning site.	This study
1Fa	ttctgaattcttaTTTATATAATTCATCCATACCATGTG Anneals to *msfGFP*. EcoRI overhang.	This study
1Rsg	gtatcattcagcacatgcaTCAGGTGGTGGAGGATC Anneals to *msfGFP*. Sec signal peptide overhang.	This study
2Fsg	gatcctccaccacctgaTGCATGTGCTGAATGATAC Anneals to Sec signal peptide sequence. *msfGFP* overhang.	This study
2Ra	ttctggtaccATGAAAAAATGTATTAAAACATTATTTTT Anneals to Sec signal peptide sequence. KpnI overhang.	This study
1Rtg	gtgttgcaattggtgcaTCAGGTGGTGGAGGATC Anneals to *msfGFP*. Tat signal peptide sequence overhang.	This study
2Ftg	gatcctccaccacctgaTGCACCAATTGCAACAC Anneals to Tat signal peptide sequence. *msfGFP* overhang.	This study
2Rb	ttctggtaccATGACAAATTATGAACAAGTTAATGA Anneals to Tat signal peptide sequence. KpnI overhang.	This study
1Rc	ttctggtaccatgTCAAAAGGTGAAGAATTATTTAC Anneals to *msfGFP* (excluding linker). KpnI restriction site.	This study
MutF	cctcctCATCAAGCTTATTTTAATTATACTC Mutagenic primer containing Shine-Dalgarno sequence.	This study
GfpR	GTACCATGAAAAAATGTATTAAAAC Reverse mutagenic primer for *msfGFP* control.	This study
SecR	GTACCATGACAAATTATGAAC Reverse mutagenic primer for *sec:msfGFP*.	This study
TatR	GTACCATGAAAAAATGTATTAAAAC Reverse mutagenic primer for *tat:msfGFP*.	This study
Coa:msfGFP_F	actaaagggaacaaaagctgggtacGGTACCGCCAAGTGAAAC Anneals to Coa:msfGFP construct. pIMAY overhang for Gibson Assembly.	This study
Coa:msfGFP_R	tcgacctcgagggggggcccggtacGGTACCAAATTTTATGAATCGAAG Anneals to Coa:msfGFP construct. pIMAY overhang for Gibson Assembly.	This study
IM151	TACATGTCAAGAATAAACTGCCAAAGC Anneals to pIMAY multiple cloning site.	[50]
IM152	AATACCTGTGACGGAAGATCACTTCG Anneals to pIMAY multiple cloning site.	[50]
OutF	GTGAAATATAGAGATGCTGGTACA Forward primer for screening *coa:msfGFP* integration	This study
OutR	TGAAGTAGGCTGAAGTTGAAGC Reverse primer for screening *coa:msfGFP* integration	This study
coa Out F	GTGCGTATAGCGGATTTTGC	This study
coa A	GGGGGTCGACGTGCGCAGCTAAAATATCGCG	This study
coa B	CCTCCAAAATGTAATTGCCCAATC	This study
coa C	GATTGGGCAATTACATTTTGGAGGTCTATCCAAAGACATACAGTCAA	This study
coa D	GGGGAGCTCGCGGGTTGAAGCAATTTCGTTT	This study
coa Out R	CGTTAGGTTATTGAATGAAGTAGG	This study
vwb Out F	GCGAGTGATTCAGACTCAGGTAGTG	This study
vwb A	GGGGGCGGCCGCGATTCAACGAGTGACACAGGATCAG	This study
vwb B	CCTTACACCCTATTTTTTCGCCAAGCC	This study
vwb C	GGCTTGGCGAAAAAATAGGGTGTAAGGGGCTGCAAAGCAAATAATGAGTTTGTCG	This study
vwb D	GGGGGCGGCCGCGTCAACACTCTCTGTCACTGATGC	This study
vwb Out R	CTAGCTGCCGATGAATCTACAATCTTATTC	This study

### Insertion of Shine-Dalgarno sequence via site directed mutagenesis

In order to make the translation of *msfGFP* possible, the Shine-Dalgarno sequence was inserted upstream of the signal peptide and *msfGFP* sequences via site directed mutagenesis [46]. The consensus sequence was chosen [47] and inserted 5 nucleotides upstream of the start codons of *tat:msfGFP, sec:msfGFP*, and *msfGFP* to ensure maximum translation efficiency [48]. To do this, a mutagenic primer, MutF, was designed with an overhang containing the Shine-Dalgarno sequence and used to amplify the entire pRMC2 constructs containing *tat:msfGFP*, *sec:msfGFP* and *msfGFP* and simultaneously insert the sequence at the desired place. A unique reverse primer was designed for each construct, while the mutagenic primer MutF remained the same (MutF/TatR for *tat:msfGFP*, MutF/SecR for *sec:msfGFP*, and MutF/GfpR for *msfGFP*). The primers were phosphorylated using T4 Polynucleotide Kinase (EK0031, Thermo Fisher Scientific) according to the manufacturer's instructions. The constructs were then amplified using the phosphorylated primers and Phusion polymerase (Phusion Hot Start II DNA Polymerase, F549S, Thermo Fisher Scientific) according to the manufacturer's instructions. The new PCR products were digested with DpnI to remove methylated template DNA, after which the mutated plasmids were ligated back into a whole plasmid according to the manufacturer's instructions (Phusion Site-Directed Mutagenesis Kit, F541, Thermo Fisher Scientific).

### Transformation into *E. coli* IM08B

The pRMC2 constructs expressing Tat:msfGFP, Sec:msfGFP, or msfGFP, and empty pRMC2, were first transformed via heat shock into *E. coli* IM08B in order to gain a methylation profile mimicking *S. aureus* [49]. To prepare chemical competent cells, an overnight culture of *E. coli* IM08B was diluted to OD_600_ 0.02 and grown to OD_600_ 0.3, then chilled on ice for 10 minutes. Cells were harvested by centrifugation at 4000 x *g* for 10 minutes at 4°C and resuspended in 5 ml ice cold 0.5 M CaCl_2_. The centrifugation was repeated, and the cells resuspended in 1.2 ml 0.5 M CaCl_2_ before incubating on ice for 30 minutes. For transformation, 1-3 µl of each pRMC2 construct was incubated for 30 minutes on ice with 50 µl of competent cells. A heat shock was applied at 4 °C for 90 s, and cells were then transferred to ice for 2 minutes. 950 µl of preheated LB media (37°C) was added and then cells incubated with 180 rpm shaking for 1 hour at 37°C. Cells were finally plated on agar with Amp and incubated at 37°C overnight. Plasmids were extracted from positive transformants with the GeneJET Plasmid Miniprep Kit (K0502, Sigma-Aldrich) and sent for sequencing with Macrogen Europe with primers FwdRMC2/RevRMC2.

### Transformation into *S. aureus* 29213

Plasmids with the correct sequence were transformed into *S. aureus* 29213 by electroporation. To prepare electrocompetent cells, an overnight culture was diluted to OD_600_ 0.5 and grown to OD_600_ 0.6. Cells were harvested by centrifugation at 4000 x *g* for 10 minutes at 4°C and washed in 50 ml ice cold MilliQ water three times. Cells were then centrifuged and resuspended in 50 ml, then 5 ml, 2 ml, and finally 0.25 ml ice cold 0.5 M sucrose. Up to 1 µg plasmid DNA was incubated on ice with 50 µl fresh competent cells for 10 minutes before being transferred to a chilled 1 mm electroporation cuvette and electroporated at 2.1 kV, 200 Ω, and 25 µF in an ECM 630 BTXTM (Harvard Apparatus). Immediately afterwards, 1 ml preheated BHI supplemented with 0.5 M sucrose (37°C) was added to the cells, which were then incubated at 37°C with 150 rpm shaking for 2 hours. Cells were finally plated on agar containing Cm and incubated overnight at 37°C. Positive transformants were confirmed by sequencing as described in the prior section.

### Creation of gene deletion mutants

In-frame single deletions of the *coa* and *vwbp* genes were achieved through splicing by overlap extension PCR according to Monk and colleagues [50] and performed as described in detail in Wassmann *et al.* 2022 [51]. The double mutant was created by introducing the pIMAYΔ*coa* plasmid into the Δ*vwbp* mutant and deleting the *coa* gene in the Δ*vwbp* mutant.

### Construction and evaluation of a chromosome-integrated Coa:msfGFP fusion protein

A C-terminal, chromosome-integrated fusion Coa:msfGFP was created by allelic replacement using the protocol from Monk *et al.* [50]. Primers Coa:msfGFP_F/Coa:msfGFP_R were used to amplify *coa:msfGFP* from a pUC57-*msfgfp* and add overhangs for Gibson Assembly. pIMAY was digested using restriction enzyme KpnI (R3142S, New England Biolabs) and then ligated to *coa:msfGFP* via Gibson Assembly [52] using a kit (E5510S, New England Biolabs). The ligated construct was first transformed via chemical transformation into *E. coli* IM08B to gain a methylation profile mimicking that of *S. aureus* [49], and was then extracted and transformed via electroporation into *S. aureus* 29213 wildtype and Δ*vwbp*, a mutant lacking vWbp, the other coagulase that *S. aureus* produces [37]. After transformation into *S. aureus*, the plasmid was then integrated into the chromosome and finally the backbone was excised using the protocol from Monk *et al.* [50]. The genotype of the fusion protein was assessed via sequencing with the OutR/OutF primers and the phenotype was assessed via coagulation assays. For coagulation assays, overnight cultures of the mutant and parental strains were diluted to OD_600_ 0.5 in 1 ml of 1:6 heparin-stabilised human plasma in 0.8 % NaCl (w/v) (S5886, Sigma-Aldrich) in sterile glass tubes and incubated for 24 hours at 37°C with no shaking. Coagulation was assessed by tilting and observing the tubes after 24 hours incubation. A negative control without bacteria was also included in addition to the mutant lacking both Coa and vWbp, which should not coagulate plasma.

### Screening for msfGFP fluorescence in cell cultures and supernatants by bulk fluorescence

To verify if msfGFP was successfully secreted by the Tat and Sec pathways, the supernatants of bacteria expressing the signal peptide and msfGFP fusions were investigated for fluorescence by bulk and in-gel measurements. Bacterial cultures and supernatants from *S. aureus* 29213 expressing Tat:msfGFP, Sec:msfGFP, msfGFP, or no msfGFP from the overexpression vector pRMC2 were grown overnight in BHI and diluted to OD_600_ 0.1 in mM9 medium and incubated at 37 °C with 180 rpm shaking until OD_600_ 0.5. mM9 was used in place of BHI because it is less autofluorescent. ATc (340 ng/ml) was added to the cultures, after which they were further incubated for 60 minutes to induce the P_xyl/tetO_ promoter and msfGFP expression. Final OD_600_ was recorded, and 2 ml of each sample taken. For the fluorescence bulk measurements, 200 µl was added directly into a 96-well plate (Nunc F96 MicroWell Black-bottom plate, 237105, Thermo Fisher Scientific) and the remaining 1.8 ml was centrifuged at 14104 x *g* for 10 minutes. The supernatant was removed, sterile filtered with a 0.2 µm filter (83.1826.001, Sarstedt), and 200 µl was added to the 96-well plate. Three biological replicates (from independently grown cultures) and three technical replicates (individually prepared samples from the same culture) were tested per construct. Fluorescence was measured at 488 nm wavelength excitation, 510 nm wavelength emission, and 1000 ms exposure time in a Varioskan Lux Flash Plate Reader (Thermo Fisher Scientific). Median fluorescence values were calculated and normalised to the optical density. The values were tested for normality with a Shapiro-Wilk test, after which they were compared using a one-way ANOVA followed by a Tukey's test with a p < 0.05 significance level.

### Visualisation of secreted msfGFP and Coa:msfGFP via in-gel fluorescence

To verify whether Sec:msfGFP and Tat:msfGFP were secreted and folded correctly, and to confirm whether Coa:msfGFP was secreted as an intact, functionally fluorescent protein, we separated the supernatant on a native PAGE gel. Cultures that expressed either Coa:msfGFP, Tat:msfGFP, msfGFP, an empty vector were grown to the exponential phase in mM9, and the chromosome-integrated Coa:msfGFP cultures were grown to the stationary phase in BHI, after which the supernatant was collected and either stored at –80°C or used right away. The supernatants were mixed 1:1 with a native sample buffer (1610738, Bio-Rad) and separated on a 4–15% precast polyacrylamide protein gel (Mini-PROTEAN TGX, 4561086, Bio-Rad). Fluorescence was detected in the Amersham Typhoon Scanner (29187191, Cytiva) with a 488 nm excitation and 510 nm emission. A His-tagged GFP (14-392, Sigma-Aldrich) was also loaded on the gel and used as a positive control for GFP fluorescence as well as a size marker.

### Confocal microscopy of *S. aureus* expressing signal peptide fusions

To visualise whether msfGFP was retained within cells, all overexpression constructs were also imaged with confocal laser scanning microscopy (CLSM). Overnight cultures were diluted to OD_600_ 0.1 in mM9 medium and were grown to OD_600_ 0.5, then incubated for a further 2 hours with 340 ng/ml ATc and imaged with the LSM700 confocal microscope (Zeiss) with a 10 mW 488nm wavelength laser at 2% power and a Plan-Apochromat 63x/1.40 NA oil immersion objective lens. Images were captured with the Axiocam HR camera (Zeiss) and using the Zen Black software (Zeiss).

### Confocal microscopy of *S. aureus* expressing Coa:msfGFP

*S. aureus* expressing either the chromosome-integrated fusion Coa:msfGFP or unmodified Coa were grown overnight in BHI and then diluted to OD_600_ 5. Microwells (µ-Slide 8 Well, 80821, IBIDI) were preconditioned with 180 µl BHI supplemented with 50% plasma, 10 µM Syto41 (S11352, Invitrogen), and 0.4 µg/ml Alexa Fluor 647-conjugated fibrinogen (F35200, Invitrogen) by incubating at 37°C for 30 minutes. Then 20 µl OD_600_ 5 cultures were added and incubated for a further 2 hours. The biofilms were imaged with 405 nm, 488 nm, and 639 nm wavelength excitation and a Plan-Apochromat 63x/1.40 NA oil immersion objective in the LSM700 confocal microscope (Zeiss). Images were captured with the Axiocam HR camera (Zeiss) and using the Zen Black software (Zeiss). GFP fluorescence was detected with 488 nm wavelength excitation and 490-600 nm wavelength emission, and Alexa 647-conjugated fibrinogen was detected with 639 nm wavelength excitation and 640-750 nm emission.

## SUPPLEMENTAL MATERIAL

Click here for supplemental data file.

All supplemental data for this article are available online at www.microbialcell.com/researcharticles/2023a-evans-microbial-cell/.

## Abbreviations:

Coa: – coagulase,
GFP: – green fluorescent protein,
msfGFP: – monomeric superfolder GFP,
Sec: – Secretory pathway,
Tat: – Twin Arginine Translocation pathway,
vWbp: – von Willebrand factor binding protein.
