# Supplementary material for: GFP fusions of Sec-routed extracellular proteins in Staphylococcus aureus reveal surface-associated coagulase in biofilms
Source: Microb Cell. 2023 Jun 28;10(7):145–56. doi: 10.15698/mic2023.07.800 (PMC10311078; doi:10.15698/mic2023.07.800)
Supplement: Supplementary file 1 [file mic-10-145-s01.pdf]

Supplemental information - GFP fusions of Sec-routed extracellular proteins in *Staphylococcus aureus* and reveals surface-associated coagulase in biofilms

Table S1 Sequences relating to the fusion proteins in this study.

Name	Sequence (5’ to 3’)	
Shine-Dalgarno	aggagg	[47]
Tat signal peptide	atgacaaattatgaacaagttaatgattcaacacaattttcacgtcgtacatttttaaaaatgttaggtattggtgg<br>tgcaggtgttgcaattggtgca	[18]
Sec signal peptide	atgaaaaaatgtattaaaacattatttttatcaattattttagttgttatgtcaggttggtatcattcagcacatgca	[45]
Linker	tcaggtggtggagga	
msfGFP	tcaaaaggtgaagaattatttacaggtgttgttccaattttagttgaattagatggtgatgttaatggtcataaattt<br>tcagttcgtggtgaaggtgaaggtgatgcaacaaatggtaaattaacattaaaatttatttgtacaacaggtaaa<br>ttaccagttccatggccaacattagttacaacattaacatatggtgttcaatgtttttcacgttatccagatcatatg<br>aaacaacatgatttttttaaatcagcaatgccagaaggttatgttcaagaacgtacaatttcatttaaagatgatg<br>gtacatataaaacacgtgcagaagttaaatttgaaggtgatacattagttaatcgtattgaattaaaaggtattga<br>ttttaaagaagatggtaatattttaggtcataaattagaatataattttaattcacataatgtttatattacagcagat<br>aaacaaaaaaatggtattaaagcaaattttaaaattcgtcataatgttgaagatggttcagttcaattagcagatc<br>attatcaacaaaatacaccaattggtgatggtccagttttattaccagataatcattatttatcaacacaatcaaaa<br>ttatcaaaagatccaaatgaaaaacgtgatcatatggttttattagaatttgttacagcagcaggtattacacatg<br>gtatggatgaattatataaa	[34]
